# Dexamethasone fails to improve bleomycin‐induced acute lung injury in mice

**DOI:** 10.14814/phy2.14253

**Published:** 2019-11-13

**Authors:** Mélissa Aubin Vega, Cécile Chupin, Mihai Pascariu, Anik Privé, André Dagenais, Yves Berthiaume, Emmanuelle Brochiero

**Affiliations:** ^1^ Centre de recherche du Centre hospitalier de l’Université de Montréal (CRCHUM) Montréal Québec Canada; ^2^ Département de Médecine Université de Montréal Montréal Québec Canada; ^3^ Institut de Recherches Cliniques de Montréal (IRCM) Montréal Québec Canada

**Keywords:** Acute lung injury, acute respiratory distress syndrome, bleomycin, dexamethasone, edema, inflammation, repair

## Abstract

Acute respiratory distress syndrome (ARDS) features an exudative phase characterized by alveolar damage, lung edema and exacerbated inflammatory response. Given their anti‐inflammatory properties, the potential therapeutic effect of corticosteroids has been evaluated in ARDS clinical trials and experimental models of ALI. These studies produced contradictory results. Therefore, our aim was to investigate the effects of dexamethasone in an animal model of bleomycin‐induced acute lung injury and then to determine if the lack of response could be related to an impairment in repair ability of alveolar epithelial cells after injury. NMRI mice were challenged with bleomycin and then treated daily with dexamethasone or saline. Bronchoalveolar lavages (BAL) and lungs were collected for assessment of the inflammatory response and wet/dry ratio (lung edema) and for histological analyses. The effect of bleomycin and dexamethasone on wound repair was also evaluated in vitro on primary alveolar epithelial cell (ATII) cultures. Our data first showed that dexamethasone treatment did not reduce the weight loss or mortality rates induced by bleomycin. Although the TNF‐*α* level in BAL of bleomycin‐treated mice was reduced by dexamethasone, the neutrophil infiltration remained unchanged. Dexamethasone also failed to reduce lung edema and damage scores. Finally, bleomycin elicited a time‐ and dose‐dependent reduction in repair rates of ATII cell cultures. This inhibitory effect was further enhanced by dexamethasone, which also affected the expression of *β*3‐ and *β*6‐integrins, key proteins of alveolar repair. Altogether, our data indicate that the inability of dexamethasone to improve the resolution of ALI might be due to his deleterious effect on the alveolar epithelium repair.

## Graphical abstract

Our data indicates that the inability of dexamethasone to improve the resolution of bleomycin outcomes, in particular, mortality rates, neutrophil infiltration and lung edema, may be due to the remaining alveolar damage observed at day 7 and repair impairment induced by dexamethasone.

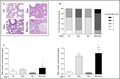

## Introduction

Acute respiratory distress syndrome (ARDS) (Ranieri et al. 2012; Fitzgerald et al. 2014), a severe form of respiratory failure, remains one of the leading causes of mortality both in adults and children in intensive care units. Various disorders, either direct (e.g., pneumonia, gastric content aspiration) or indirect (e.g., sepsis, trauma), are associated with the development of ARDS (Monahan 2013; Ware and Matthay 2000). However, regardless of the causes, ARDS features overlapping exudative, proliferative and fibrotic phases. The acute exudative phase is characterized by extensive alveolar epithelial and endothelial damage, eliciting alveolar edema, neutrophil infiltration, high levels of chemokines/cytokines and decreased lung compliance (Ware and Matthay 2000; Monahan 2013). Collagen deposition and fibroproliferation, competing with epithelial repair, can rapidly progress toward irreversible pulmonary fibrosis, ultimately leading to respiratory failure (Ware and Matthay 2000; Shimabukuro et al. 2003; Ranieri et al. 2012). Therefore, the resolution of the acute phase is pivotal for ARDS recovery.

Although improvements in mechanical ventilation procedures have been associated with increased survival over the last decades (Ranieri et al. 2012; Fitzgerald et al. 2014), mortality rates (30–45%) still remain unacceptably high, and effective, noninvasive pharmacological therapies are needed. Because the inflammatory response is a key determinant of ARDS, several studies have evaluated the efficiency of anti‐inflammatory therapies. One of the most studied therapy has been systemic corticosteroids. Clinical trials on ARDS patients and subsequent meta analyses (Steinberg et al. 2006; Foster 2010; Yehya et al. 2015; Meduri et al. 2016; Kimura et al. 2016; Tongyoo et al. 2016; Standiford and Ward 2016; Yang et al. 2017; Fan et al. 2018) as well as experimental studies in animal models of acute lung injury (ALI) (Chen et al. 2006; Leite‐Junior et al. 2008; Wang et al. 2008; Xu et al. 2009; Yubero et al. 2012; Hegeman et al. 2013; Engel et al. 2015) have indicated that glucocorticoids (e.g., methylprednisolone, dexamethasone, hydrocortisone) elicit variable effects as a function of the dose, the route of administration, the time to treatment initiation and duration, the age of the patient, the cause of ARDS/type of ALI model and/or the measured outcomes. Therefore, the available evidence for glucocorticoids benefits in ARDS is conflicting and the use of these treatments in ARDS patients are still subject to debate (Bein et al. 2016; Bihari et al. 2016; Seam and Suffredini 2016; Thompson and Ranieri 2016; Mac and McAuley 2017; Meduri and Siemieniuk 2017; Bos et al. 2018).

The goal of our study was first to dissect the effect of daily treatments with dexamethasone in an experimental model of ALI in mice. We opted for the well‐characterized model of bleomycin‐induced lung injury and focused our study on the acute exudative phase, featuring an endothelial/alveolar epithelial damage, lung edema, neutrophil infiltration and decreased lung function (over a 7‐day period), before establishment of fibrosis (day‐12–21) (Matute‐Bello et al. 2008; Saito et al. 2008; Goto et al. 2010). The effect of the dexamethasone treatment was evaluated by assessing mouse survival, lung edema, inflammatory response and alveolar injury after the bleomycin challenge. Our data indicated that dexamethasone reduced TNF‐*α* levels but failed to improve mouse survival or to reduce lung edema, neutrophil infiltration and injury scores after bleomycin. We then hypothesized that dexamethasone may impair the repair ability of alveolar epithelial cells after injury. We thus analyzed the effect of bleomycin and dexamethasone in vitro on the wound healing of primary alveolar epithelial cell cultures and showed that dexamethasone worsened the deleterious effect of bleomycin on the repair rates.

## Materials and Methods

### In vivo experimental design

Wild‐type NMRI mice (Naval Medical Research Institute, Bethesda, Maryland, USA) were kindly gifted by E. Hummler (Lauzanne, Switzerland). All procedures were conducted according to the Canadian Council on Animal Care (CCAC), and the experimental protocol was approved by the Institutional Animal Protection Committee (CIPA) of the Centre de Recherche du Centre hospitalier de l'Université de Montréal (CRCHUM). Animals were sheltered under standard conditions with food and water provided ad libitum. Experiments were conducted on 7‐ to 10‐week‐old male mice, randomly divided into 4 groups: Ctl (control, instillation of saline and daily intra‐peritoneal (i.p.) treatment with saline), Bleo (instillation with bleomycin and daily i.p. treatment with saline), Dex (instillation with saline and daily i.p. treatment with dexamethasone) and Bleo + Dex (instillation with bleomycin and daily i.p. treatment with dexamethasone). More precisely, mice were anesthetized at day 1 with a solution (0.01 mL/g) of 13% ketamine (100 mg/mL) and 1.3% xylazine (20 mg/mL) in 0.9% saline. Then, animals were instilled intratracheally (i.t.) with saline (0.9%, 50 *µ*L) or bleomycin (MaynePharma Canada, QC, Canada, 4 U/kg, 50 *µ*L) following a modified non‐surgical and non‐damaging method (Guilbault et al. 2005). Immediately after instillation of saline or bleomycin, mice were treated with dexamethasone (Sandoz Canada, QC, Canada, 0.5 mg/kg, 100 *µ*L) or vehicle (saline 0.9%, 100 *µ*L) by i.p. Treatments (saline or dexamethasone) were repeated daily and outcomes (see below) were measured 3, 7 and/or 12 days after the bleomycin challenge, corresponding to the development of acute lung injury, as described in the literature (Matute‐Bello et al. 2008; Saito et al. 2008; Goto et al. 2010). As supplementary experiments (Fig. S1), a group was treated with methylprednisolone (NovoPharm, QC, Canada, 1 mg/kg, 100 *µ*L, daily, i.p. treatment).

### Mice survival rates and weight variation

The mice weight variations were calculated from the measured weights at each time point (days 3, 7 and 12), including before sacrifice of mice reaching the endpoints (see below), and reported as % of the initial weight before the bleomycin challenge. In compliance with the CCAC standards and following daily animal assessment by the CRCHUM animal care personnel, mice reaching endpoints (respiratory failure, prostration, uncontrollable pain, dehydration, or loss of more than 30% of the initial weight), were sacrificed according to the procedure approved by the CRCHUM institutional animal care committee. To avoid a potential bias by studying the animals with the better outcomes, animals reaching endpoints were included in the calculation of the weight variation and survival rates (which were reported as % of the live mice in each group at 3, 7 and 12 days after the beginning of the bleomycin challenge). Subsequent experiments, for the measurement of lung edema, tissue injury and inflammatory response (see below), were performed during the acute exudative phase (day 3 and 7). It has to be noted that at these time points no (day 3) or small (day 7, <10%) mortality rates were observed.

### Edema index (wet/dry ratio)

After euthanasia (with 0.02 mL/g, ketamine‐xylazine) on days 3 and 7, the inferior *vena cava* was severed, the lungs were removed and directly weighed (wet weight). Lungs were heated to 95°C for 24 h to measure the dry weight and then to calculate the wet/dry ratio.

### Bronchoalveolar lavages (BAL)

In another series of experiments, BAL were performed after mouse euthanasia (at days 3 and 7, in each condition) by instillation of saline (1 mL) through a catheter and then gentle aspiration. Six repeated BAL (from the same mouse) were collected and pooled on ice before centrifugation (200*g*, 4°C, 8 min). The supernatants were stored at −80°C until subsequent use to determine protein concentration and TNF‐*α* levels.

The protein concentration in BAL supernatants was evaluated by the Bradford method (Bio‐Rad Life Science, Mississauga, ON, Canada). TNF‐*α* levels in BAL supernatant samples were measured by AlphaLISA technology (AL505 C/F, PerkinElmer, Montreal, QC, Canada). Following the manufacturer’s recommendation, experiments were performed in triplicate at room temperature, and TNF‐*α* concentrations (pg/mL) were estimated from a TNF‐*α* standard curve (dynamic range from 2.0 to 30,000 pg/mL) after reading with the EnVision‐Alpha Reader (PerkinElmer).

Cell pellets were resuspended in 500 *µ*L of PBS for quantification of the total cell count. The cell suspensions were then diluted at a density of 1 × 10^6^ cells/mL, cytocentrifuged (300 rpm, 3 min, Shandon Cytospin 3 Centrifuge, Block Scientific, NY, USA) onto glass slides (4 × 10^4^ cells/slide) and stained with Hema‐3^®^ (Fisher, US). The differential cell count (number of neutrophils, macrophages, lymphocytes and eosinophils (reported as percentage) among a total of 400 leukocytes) was then determined.

### Histological analysis and lung damage severity scores

Mice lungs, collected 7 days after the initial bleomycin challenge, were fixed by immersion in a 10% formalin solution, embedded in paraffin and the sections were stained with hematoxylin and eosin according to standard protocols at the Institut de Recherche en Immunologie et en Cancérologie (IRIC, Université de Montréal). Blind histological analysis was performed by Dr. Louis Gaboury (pathologist, Histology and Molecular Pathology research unit, University of Montréal), who defined a qualitative severity score (from 0 to 4), adapted from a well‐recognized scoring system to evaluate experimental ALI in animals (Matute‐Bello et al. 2011) and incorporating the following criteria: the presence of mononucleated cells/macrophages, polymorphonuclear, fibrinous exudate/hyaline membranes, widening of the septae, regenerative atypias/karyomegaly, intraalveolar hemorrhage, pneumocyte sloughing/cell debris, bronchial exudate, congestion/edema and consolidation. The results were expressed as the percentage of mice with each severity grade (from 0 to 4) of lung damage within the treatment group. Specimens were photographed using a digital camera (DP71) mounted on an Olympus BX61 microscope.

### Alveolar epithelial cell (ATII) isolation and primary culture

Alveolar epithelial cells (ATII) were isolated from adult male Sprague‐Dawley rats (6–7 weeks, Charles‐River, St‐Constant, QC) according to a well‐established protocol (Dagenais et al. 2006; Bardou et al. 2012; Girault et al. 2015). Briefly, lungs were washed with a physiological solution to remove excess blood cells and alveolar macrophages. Then, the lungs were digested with 160 U/rat elastase solution (Worthington Biochemical, Lakewood, N.J. USA) and minced, and the resulting cell suspension was filtered. Alveolar cells were purified using a differential adherence technique (Dobbs et al. 1986), which enhances the purity of the ATII cell pool by up to 86% (Brochiero et al. 2004). The freshly isolated ATII cell suspension was then seeded on 6 or 12‐well cell culture clusters (Corning) and cultured in minimum essential medium (MEM, Invitrogen, Burlington, ON, Canada) supplemented with 10% FBS (Invitrogen, Canada), 0.2% NaHCO_3_ (Sigma‐Aldrich), 0.01 mol/L HEPES (Thermo‐Fisher Scientific Inc.), 2 mmol/L l‐glutamine (Invitrogen, Canada), 0.08 mg/L gentamicin (Life Technologies) and Septra (Aspri Pharma Canada, Canada, 3 g/mL trimethoprim and 17 g/mL sulfamethoxazole) at 37°C with 5% CO_2_ in a humidified incubator. This medium was replaced by MEM + 10% FBS without Septra at day 4, as previously described (Dagenais et al. 2006; Leroy et al. 2006; Dagenais et al. 2013; Girault et al. 2015). Well clusters with ATII cells from the same animal were then randomly divided into the different experimental conditions (treatments, see below) and experiments repeated on cell cultures from at least 4 animals (as indicated in the figure legend).

### Wound‐healing assays

At day 2 of primary culture, ATII cell monolayers were treated, or not, for 24 h with dexamethasone (100 nmol/L in MEM medium supplemented with FBS and Septra (see above)). At day 3, mechanical injuries with a P10 Gilson pipette tip (6 wounds per Petri dish) were performed according to a well‐established, highly reproducible technique (Maillé et al. 2011; Ruffin et al. 2016; Adam et al. 2018). Immediately after injury (T0), the monolayers were then washed (with MEM + FBS without Septra) to remove detached cells, and the injured monolayers were treated with bleomycin (Bleo, 12.5–200 mU/mL), dexamethasone (Dex, 100 nmol/L), a combination of bleomycin and dexamethasone (Bleo + Dex) or vehicle (Ctl, saline 0.9%). Monolayers were photographed with a NIKON camera under light microscopy at T0, T24 h, T36 h and T48 h after injury. A mark under the Petri dishes allowed us to photograph the wounds at exactly the same place at every time point. After analysis with ImageJ software (NIH, Bethesda, MD, USA), the wound area was measured, and the wound healing rates (*μ*m^2^/h) were calculated. Wound healing assays on cell monolayers provide insight into the initial repair processes engaged after injury (mainly cell migration and proliferation).

### ATII cell apoptosis

ATII cells were collected after a 24 h treatment with bleomycin (Bleo, 50 mU/mL), dexamethasone (Dex, 100 nmol/L), a combination of bleomycin and dexamethasone (Bleo + Dex) or vehicle (Ctl, saline 0,9%) and then caspase‐3/7 activity was determined by luminescent assay (630/595 nm) using the Caspase Glo kit (Promega), following the manufacturer instructions.

### Immunoblotting

Freshly isolated primary ATII cells were seeded (2.5 × 10^6^ cells/well) in 6‐well cell culture clusters (Costar; Corning, Corning, NY). At day 4, ATII cell monolayers were treated with bleomycin (Bleo, 50 mU/mL), dexamethasone (Dex, 100 nmol/L), a combination of bleomycin and dexamethasone (Bleo + Dex) or vehicle (DMSO) and total proteins were extracted 24 h later. Briefly, ATII cell monolayers were washed twice with PBS, scraped with 200 *µ*L/well lysis buffer (NaCl 150 mmol/L, EDTA 5 mmol/L, TRIS 50 mmol/L, Triton X‐100 1%, pH 7.5, plus a cocktail of proteases/phosphatases inhibitors (Sigma)). The suspension was centrifuged at 13,000 rpm for 10 min at 4°C. Supernatants were collected and protein content was evaluated by the Bradford method. Sample proteins were denatured at 95°C for 10 min, separated by SDS‐PAGE (10%) and transferred to a PVDF membrane. PVDF membranes were first blocked with 5% powdered milk in Tris‐buffered saline + 0.1% Tween 20 (TBST) for 1 h at room temperature and then washed three times with TBST before overnight incubation (at 4°C) with anti‐*β*3‐integrin (Abcam, dilution 1:1000, TBST + 5% Bovine serum albumin (BSA, Sigma)), anti‐*β*6‐integrin (Santa Cruz/Millipore, dilution 1:1000, TBST + 5% BSA), anti‐*β*‐actin (Sigma, 1:1000, TBST + 5% BSA) or anti‐pan‐actin (Cell signaling, 1:1000, TBST + 5% BSA) antibodies. Membranes were then washed with TBST and incubated with horseradish peroxidase‐labeled secondary antibody (goat anti‐rabbit (Santa Cruz/Cell signaling), mouse anti‐goat (Santa Cruz) and goat anti‐mouse (Sigma/Abcam), dilution 1:1000, TBST + 5% powdered milk). Membranes were rinsed (TBST, 3 × 15 min) and incubated with a luminescent reactive Immun‐Star WesternC Kit (Bio‐Rad Laboratories Inc.) or Western Lightning Plus‐ECL (PerkinElmer). The intensity of each band was measured with a ChemiDoc system (BioRad Laboratories Inc.), quantified with the Image Lab program (BioRad Laboratories Inc.) and normalized to the actin signal. Protein expression is presented as a percentage of the control condition (100%).

### Statistical analysis

**Figure 1:**
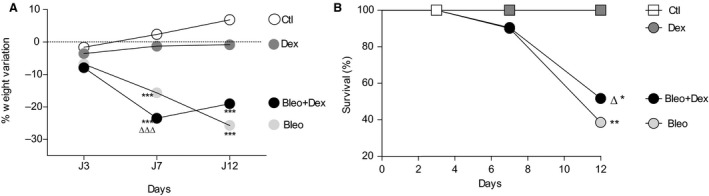
Effect of dexamethasone on body weight and survival after bleomycin‐induced acute lung injury in mice. After i.t. instillation (at day 1) of saline (0.9%, 50 *μ*L) or bleomycin (Bleo, 4 U/kg, 50 *μ*L), male NMRI mice were treated (i.p. injection) daily with saline (0.9%, 100 *μ*L) or dexamethasone (Dex, 0.5 mg/kg, 100 *μ*L) for 3, 7 or 12 days. Weight variations (A, *n* = 3–21) as a function of time and percentage (%) of mouse survival (B, *n* = 6–20) were compared between the four groups: Ctl (saline (i.t.)/saline (i.p.)); Bleo (bleo (i.t.)/saline (i.p.)); Dex (saline (i.t.)/dex (i.p.)) and Bleo + Dex (bleo (i.t.)/dex (i.p.)). Values are means ± SEM, **P < 0.01, ***P < 0.001 versus control condition at the same time point, *^∆∆^P* < 0.01, *^∆∆∆^P* < 0.001 versus Dex condition at the same time point. 1‐way ANOVA (Agostino/Pearson normality positive tested, *P* < 0.0001) and Bonferroni post hoc test (A, day 3), 1‐way ANOVA; Kruskal–Wallis test and Dunn’s post hoc test (A, day 7 and 12). Comparison of survival curves was made with a Log‐rank (Mantel‐Cox) test which generated a Chi square, **P < 0.01 Bleo versus Ctl curve, *P < 0.05 Bleo + Dex versus Ctl curve and ^∆^ *P* < 0.05 Dex versus Bleo + Dex curve (B)

Mice survival rates (Fig. 1B) are presented as percentages (%) of living mice compared to the initial group for each treatment group, while lung damage scores (Fig. 4B) are presented as a repartition of mice with each damage score (expressed as % of the total number of mice in each treatment group). All other data are presented as means ± standard error of the mean (SEM). Graphs and statistical analyses were performed with GraphPad Prism 5 software (CA, USA). Agostino/Pearson normality tests were first performed, followed by statistical tests, adapted to each type of experiment, as specified in figure legends. *P* values are also indicated for each series of experiments (*P* values < 0.05 (*) were considered significant).

## Results

While progressive weight gain was observed in the control (Ctl) group as a function of time (at days 3, 7 and 12), lung delivery of bleomycin (Bleo, 4 U/kg) caused a significant weight loss at days 7 and 12 (Fig. 1A). However, daily (i.p.) treatment with dexamethasone (0.5 mg/kg) in mice with bleomycin‐induced lung injury (Bleo + Dex group) did not prevent weight loss.

A 100% survival rate was observed in the control (Ctl) and dexamethasone (Dex) groups at each time points, whereas the bleomycin challenge (Bleo and Bleo + Dex groups) induced an increasing mortality rate (*P* < 0.05). Dexamethasone treatment (Bleo + Dex) did not significantly improve the survival (Fig. 1B).

**Figure 2:**
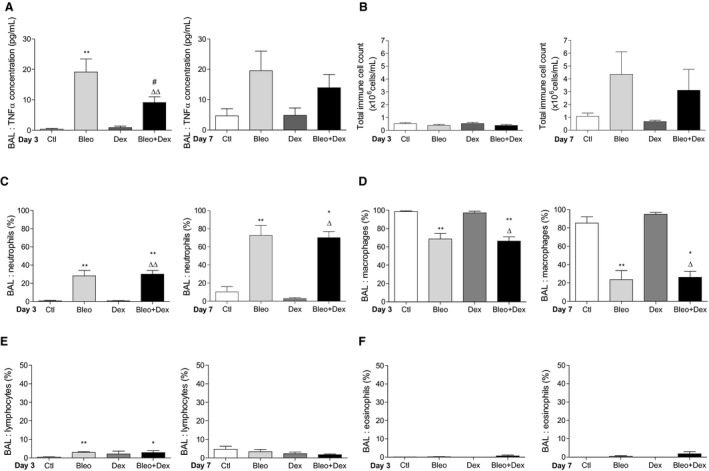
Effect of dexamethasone on the inflammatory response after bleomycin‐induced acute lung injury in mice. A. Levels of TNF‐*α* (pg/mL) detected by ELISA in BAL collected at day 3 (*n* = 6, left panel) and 7 (*n* = 5–7, right panel). Total immune cell counts (B) and differential cell count (% of neutrophils (C), macrophages (D), lymphocytes (E) and eosinophils (F)) in BAL collected from mice 3 (left panel) or 7 (right panel) days after the bleomycin challenge (4 U/kg, 50 *μ*L i.t., *n* = 4–7). Values are means ± SEM, *P < 0.05, **P < 0.01 versus Ctl condition, *^#^P* < 0.05 versus Bleo condition, *^∆^P* < 0.05*, ^∆∆^P* < 0.01 versus Dex condition. Non‐parametric *t*‐test (Mann–Whitney, panel A) or 1‐way ANOVA; Kruskal–Wallis test and Dunn’s post hoc test (panels B, C, D, E, F)

In subsequent experiments, the effect of dexamethasone on the bleomycin outcomes was measured during the acute exudative phase, i.e., at 3 and 7 days. We first evaluated the levels of the TNF‐*α* cytokine, which plays a key role in alveolar epithelial damage and dysfunction in acute lung injury (Patel et al. 2013). We found that exposure to bleomycin was associated with a significant increase in TNF‐*α* in the BAL collected at day 3 (Bleo and Bleo + dex group, Fig. 2A), and that the TNF‐*α* levels in the Bleo + Dex group were significantly lower compared to the Bleo group on day 3. The same trend was observed at day 7, although the variations were not statistically significant.

Elevated total leukocyte cell counts in BAL were observed at day 7 in the Bleo and Bleo + Dex conditions (Fig. 2B). Differential cell count then revealed that compared to that in the control group, a significant increase in the proportion of neutrophils was observed in the BAL of mice challenged with bleomycin (Fig. 2C). The proportion of neutrophils was similar in the Bleo + Dex group. This increase in neutrophils induced by bleomycin was associated with a parallel decrease in the proportion of macrophages in both the Bleo and Bleo + Dex groups (Fig. 2D). The proportion of lymphocytes and eosinophils were lower than 5% in all experimental conditions (Fig. 2E and 2F).

**Figure 3:**
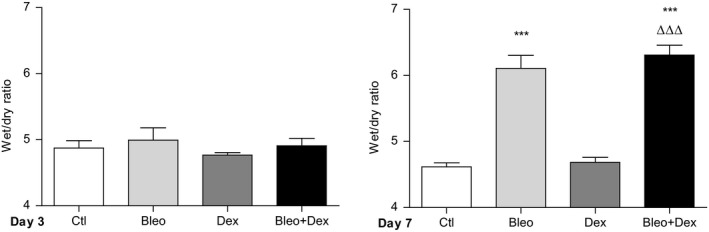
Effect of dexamethasone on edema formation after bleomycin‐induced acute lung injury in mice. Wet‐to‐dry lung weight ratios (edema index) were measured 3 (*n* = 12, left panel) and 7 (*n* = 14–22, right panel) days after initial instillation (day 1) of saline (Ctl, 0.9%) or bleomycin (Bleo, 4 U/kg) and daily treatments (i.p. administration) with saline (0.9%) or dexamethasone (Dex, 0.5 mg/kg, 100 *μ*L). Values are means ± SEM, ***P < 0.001 versus Ctl condition*, ^∆∆∆^P* < 0.001 versus Dex condition. 1‐way ANOVA; Kruskal–Wallis test and Dunn’s post hoc test

Edema flooding was also associated with the bleomycin‐induced damage of the alveolar epithelial/endothelial barriers. Indeed, at day 7 the wet/dry (W/D) ratio is significantly higher in bleomycin‐challenged mice (6.1 ± 0.2), compared to the control condition (4.6 ± 0.1) (Fig. 3, right panel). Moreover, dexamethasone treatment did not prevent bleomycin‐induced edema. Similarly to dexamethasone, another corticosteroid (methylprednisolone, Fig. S1) did not elicit any beneficial effect on lung edema in bleomycin mice.

**Figure 4:**
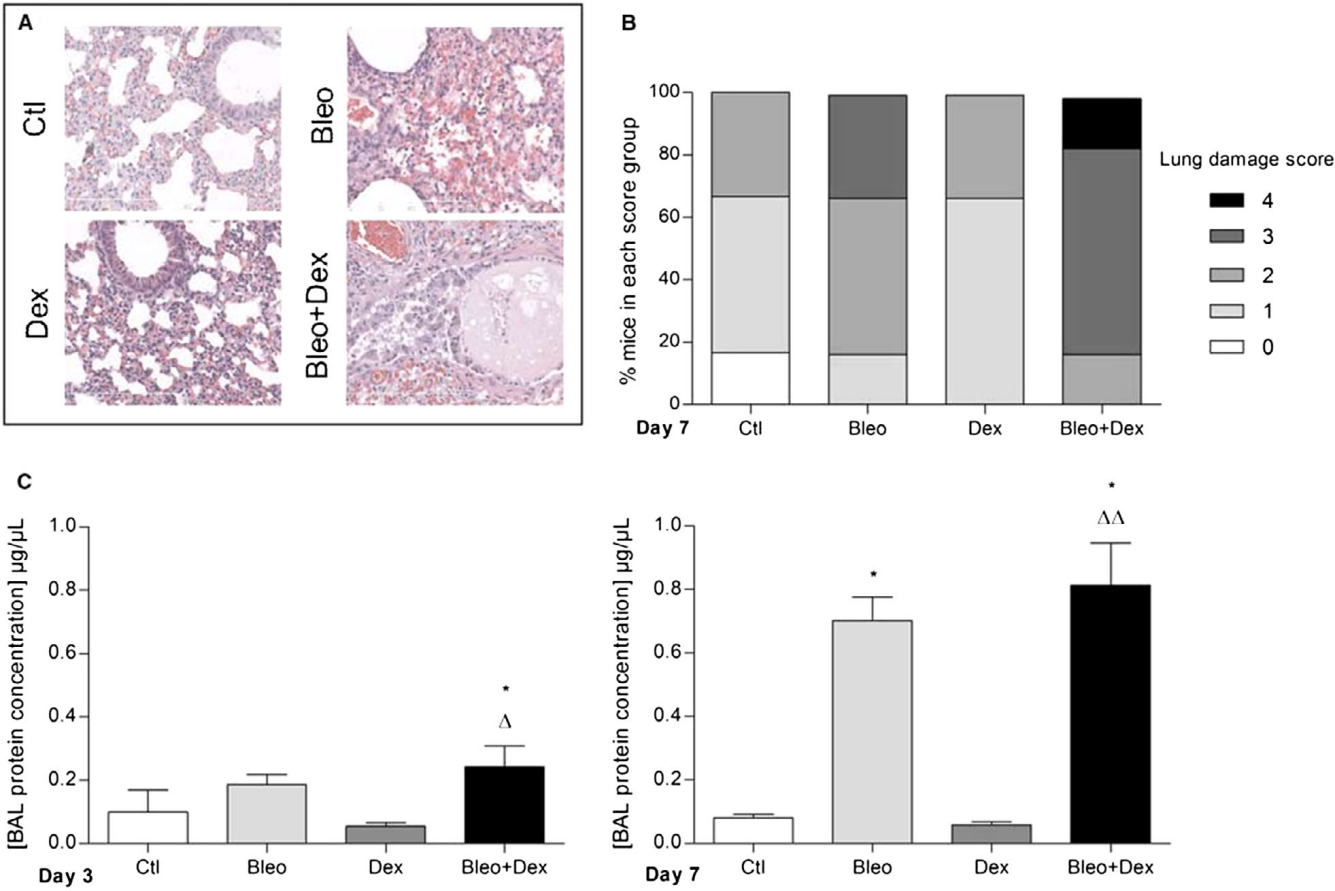
Effect of dexamethasone on alveolar epithelial damage after bleomycin‐induced acute lung injury in mice. Histological sections of mouse lungs stained with hematoxylin‐eosin (Magnification × 20. Scale: 200 µm, A) and repartition of lung damage scores at day 7 (B, *n* = 6–8) are presented for each group (Ctl (saline (i.t.)/saline (i.p)); Bleo (bleo (i.t.)/saline (i.p.)); Dex (saline (i.t.)/dex (i.p.)) and Bleo + Dex (bleo (i.t.)/dex (i.p.)). The concentration of proteins in BAL (*μ*g/*μ*L, C, *n* = 5–6) was measured at day 3 (left panel) and 7 (right panel). Values are means ± SEM, *P < 0.05 versus Ctl condition, *^∆^P* < 0.05*, ^∆∆^P* < 0.01 versus Dex condition. 1‐way ANOVA; Kruskal–Wallis test and Dunn’s post hoc test

Because the observed inability to clear the lung edema could be due to persistent alveolar damage in the presence of bleomycin and dexamethasone, histological analyses (Fig. 4) were then performed. Substantial lung injury was observed in the presence of bleomycin, with injury scores of 2 and 3 in 50% and 33.3% of mice, respectively (Fig. 4B). Mice in the Bleo + Dex group exhibited severe injury scores [2 (16.7%), 3 (66.7%) and 4 (16.7%)]. Alveolar damage was also associated with a significant increase in the protein concentration in the BAL of mice as early as day 3 in the Bleo + Dex group and at day 7 in the Bleo condition (Fig. 4C).

**Figure 5:**
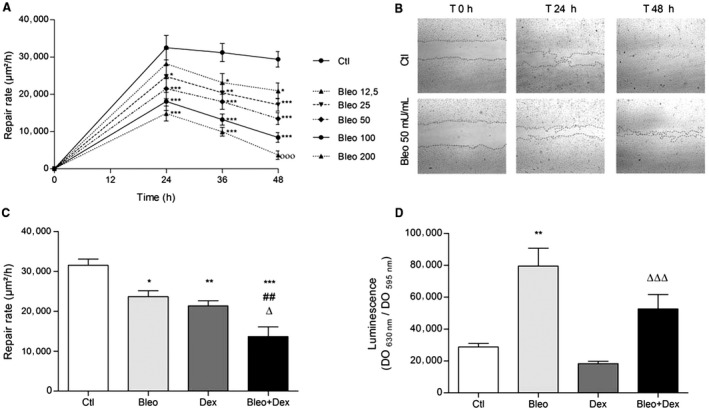
Effect of dexamethasone on the repair rates of primary ATII cell monolayers. (A) Primary rat ATII cell monolayers were injured mechanically, and repair rates (*μ*m^2^/h) were measured over periods of 24, 36 and 48 h in the control (Ctl) condition (saline, 0.9%) and after treatments with increasing doses of bleomycin (Bleo, 12.5, 25, 50, 100, 200 mU/mL, *n* = 8). (B) Representative photographs of healing ATII cell monolayers at 0, 24 and 48 h after treatment with saline (0.9%) or bleomycin (Bleo, 50 mU/mL). (C) Repair rates (*μ*m^2^/h) of ATII cell monolayers in control condition (Ctl, 0.9%), after treatment with bleomycin alone (Bleo, 50 mU/mL, applied at T0), dexamethasone alone (Dex, 100 nmol/L, applied 24 h before injury) and a combination of bleomycin (50 mU/mL, T0) and dexamethasone (100 nmol/L, 24 h before injury) (Bleo + Dex, *n* = 8). (D) ATII cell apoptosis in control condition (Ctl, 0.9% saline) and after 24 h treatment with bleomycin alone (Bleo, 50 mU/mL), dexamethasone alone (Dex, 100 nmol/L) or a combination of bleomycin (50 mU/mL) and dexamethasone (100 nmol/L) (Bleo + Dex) (*n* = 10). Values are means ± SEM, *P < 0.05, **P < 0.01, ***P < 0.001 versus Ctl condition, *^##^P* < 0.01 versus Bleo condition, *^∆^P* < 0.05 versus Dex condition. 2‐way ANOVA and Bonferroni post hoc test (vs. control at the same time point, panel A), 1‐way ANOVA (Agostino/Pearson normality positive tested, *P* < 0.0001) and Bonferroni post hoc test (panel C), 1‐way ANNOVA Kruskal‐Wallis test and Dunn’s post hoc test (panel D)

We then hypothesized that this persistent alveolar damage could be due to a deleterious effect of bleomycin and dexamethasone on the repair capacity of alveolar cells. To test this hypothesis, we then performed a series of wound healing assays on primary alveolar epithelial (ATII) cell cultures. As depicted in Figure 5A, a time‐ (over a 48 h period after injury) and dose‐ (12.5 to 200 mU/mL) dependent inhibition of the wound healing rates was observed in the presence of bleomycin. Because a dose of 50 mU/mL significantly decreased the repair rates at every time point, this concentration was used in subsequent experiments (Fig. 5B–D). Our data showed that a 24 h treatment with dexamethasone (100 nmol/L, a dose previously shown to elicit biological effects on ATII cells (Champigny et al. 1994; Dagenais et al. 2006)) alone significantly reduced the wound healing rates (21,304 ± 1328 vs. 31,523.1 ± 1519 *µ*m^2^/h in control condition) and further dampened the repair rates measured in the presence of bleomycin (23,642.9 ± 1484 *µ*m^2^/h and 13,671 ± 2416 *µ*m^2^/h in Bleo and Bleo + Dex, respectively).

A pro‐apoptotic effect of bleomycin has been established in several cell models (Lee et al. 2005; Wallach‐Dayan et al. 2006). In agreement with these previous reports, a significant increase in caspase 3/7 activity was noted in bleomycin‐treated ATII cells (in Bleo and Bleo + Dex conditions, compared to Ctl and Dex, respectively) (Fig. 5D). Although, ATII cell apoptosis was slightly lower in the presence of Dex (Bleo + Dex, compared to Bleo alone), the decrease was not statistically significant.

**Figure 6:**
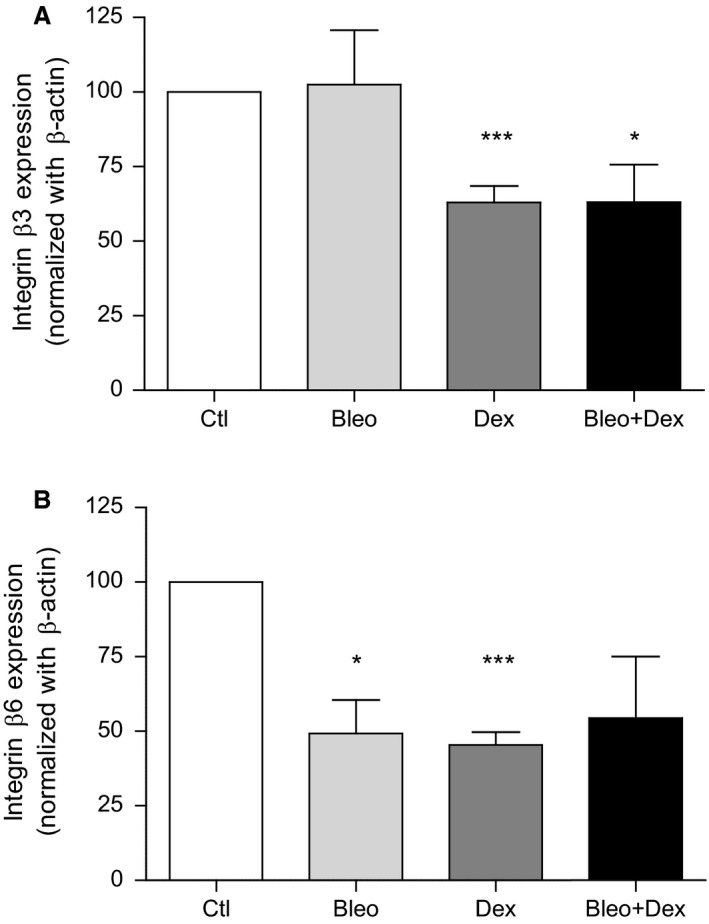
Effect of dexamethasone on *β*3‐ and *β*6‐integrin expression in primary ATII cells. Protein expression (% of control condition normalized with actin expression) of *β*3‐ (upper panel, *n* = 6–14) and *β*6‐integrin (lower panel, *n* = 3–8) in control, bleomycin, dexamethasone and bleomycin + dexamethasone pretreated (24 h) conditions. Values are presented as means ± SEM, one simple *t*‐test with a theoretical mean of 100%, *P < 0.05 and ***P < 0.001 versus Ctl condition

The negative effect of dexamethasone on wound healing was associated with a significant decrease in the expression of two key proteins of alveolar repair, i.e., *β*3‐ and *β*6‐integrins (37 and 55% decrease, compared to the control condition, when the cells were treated with dexamethasone (Fig. 6)). A significant reduction in *β*6‐integrin expression was also observed after exposure to bleomycin. The Bleo + Dex combination also induced a decrease in *β*3‐ and *β*6‐integrins (although non‐statistically significant for *β*6‐integrin).

## Discussion

Although dexamethasone treatment efficiently prevented the TNF‐*α* response after bleomycin‐induced lung injury, our experiments showed that it failed to reduce the neutrophil infiltration in BAL, weight loss and mortality rates, as well as lung edema and injury scores. Our data support the hypothesis that this inability of dexamethasone to improve the resolution of bleomycin outcomes may be due to its damaging effect on the repair capacity of the alveolar epithelium.

Our data first showed that daily treatments with dexamethasone over a 12‐day period failed to prevent weight loss associated with the inflammatory response after the bleomycin challenge. The muscle atrophy induced by glucocorticoids might also be responsible for this inability to gain weight (Schakman et al. 2009; Bodine and Furlow 2015). Our study also demonstrated that the number of mice requesting euthanasia because they reached endpoints was similar in the Bleo and Bleo + Dex experimental groups. These findings are in agreement with previous reports indicating that dexamethasone did not reduce weight loss and/or mortality rates in experimental models of acute lung injury (Koshika et al. 2005; Xu et al. 2009).

Our results also show that although dexamethasone treatment efficiently dampened the early TNF‐*α* increase (at day 3), it did not impair the inflammatory response or the injury process. Indeed, the increase in neutrophils, the elevated protein content measured in the BAL, the injury score and the increase in the wet/dry lung weight ratio in the presence of bleomycin on day 7 were not attenuated by dexamethasone, at the tested dose. This persistent neutrophil infiltration most likely contributes to the epithelial injury (Ware and Matthay 2000; Grommes and Soehnlein 2011) observed in our histological analyses. This inability of dexamethasone to protect/improve the alveolar barrier has also been observed in other models of severe acute lung injury (Xu et al. 2009; Yubero et al. 2012; Hegeman et al. 2013; Engel et al. 2015). Indeed, our results are in phase with the variable response observed following dexamethasone treatment of lung‐injured animals. In agreement with our findings, reduced TNF‐*α* levels were observed in bleomycin + dexamethasone‐treated rats compared to those in the bleomycin condition (Yang et al. 2015). Yubero et al. also reported that intramuscular injection of dexamethasone (1 mg/kg), 1 h after induction of lung injury due to acute pancreatitis in rats, downregulated inflammatory factors but did not reduce leukocyte infiltration (Yubero et al. 2012). However, i.v. administration of dexamethasone at the initiation of ventilation‐induced lung injury in mice has been shown to attenuate both inflammatory mediator expression (KC, MCP‐1, IL‐1*β*, IL‐6) in lung tissues and neutrophil infiltration in BAL (Hegeman et al. 2013). In contrast, neither cellular infiltration nor cytokine release were inhibited by dexamethasone in a model of acute lung injury induced by the H5N1 virus in mice (Xu et al. 2009) and in ventilated adult sheep with early phase acute respiratory distress syndrome (Engel et al. 2015). Although the variability observed is in part secondary to the outcomes that are measured, the administration of dexamethasone alone does not appear to be efficient in reversing the evolution of lung injury or counteracting its undesirable side effects (Xu et al. 2009; Kohno et al. 2010; Yubero et al. 2012; Hegeman et al. 2013; Engel et al. 2015).

The persistent lung edema and/or inflammatory cell infiltration observed in our model and others (Xu et al. 2009; Yubero et al. 2012; Hegeman et al. 2013; Engel et al. 2015), despite the presence of dexamethasone, may be due to severe and nonresolving alveolar damage, at the measured time points. In agreement with our hypothesis of a deleterious impact of bleomycin and dexamethasone on the repair capacity of the alveolar epithelium, our experiments first show a time‐ and dose‐dependent inhibition of wound repair rates of primary ATII cell cultures by bleomycin. The observed pro‐apoptotic effect of bleomycin, could contribute, at least in part, to this repair impairment. Not only did dexamethasone not reverse the bleomycin‐repair impairment, but it further worsened the repair delay in vitro as well as the injury scores and protein levels in BAL in vivo. In agreement with our results, a deleterious effect of dexamethasone on repair mechanisms has also been shown in other epithelial cells (Liu et al. 2013; Kadmiel et al. 2016). Furthermore, it has been shown that dexamethasone inhibits corneal epithelial wound healing and cell migration (by altering the activity of membrane lamellipodia and filopodia) but promotes tight junction integrity (Kadmiel et al. 2016). The role of corticosteroid‐induced apoptosis on airway epithelial repair impairment has also been studied. A previous study by Dorscheid et al (2006) indicated that the observed airway epithelial cell apoptosis induced by dexamethasone or budesonide was not involved in the decrease in wound repair rates after corticosteroid exposure. At lower concentration, dexamethasone elicited in our study a slight, but non‐significant, decrease in caspase 3/7 activity in ATII cells in the absence or presence of bleomycin.

The decreased *β*3‐ and *β*6‐integrin expression that we observed in alveolar epithelial cells may be involved in dexamethasone‐induced repair impairment. However, other mechanisms may also be involved. Indeed, a previous study indicated that the inhibition of airway epithelial repair, cell proliferation and migration by dexamethasone may be mediated, at least in part, by suppression of the MAPK/ERK signaling pathway (Liu et al. 2013). Similarly, a marked inhibition in cell proliferation and migration, associated with decreased levels of Mek1/2‐p‐Erk1/2, has been observed in human airway epithelial cell (16HBE) cultures in the presence of dexamethasone (Jia et al. 2018).

## Conclusions

Altogether, our data indicates that the inability of dexamethasone to improve the resolution of bleomycin outcomes, in particular, mortality rates, cell infiltration and lung edema, may be due to the remaining alveolar damage observed at day 7 and repair impairment induced by dexamethasone. Although data from animal models should be taken with caution and never perfectly reflect the physiopathology of ARDS, our study and those previously published suggest that dexamethasone alone is unlikely to be an efficient therapy for acute lung injury. Indeed, there is accumulating evidence that successful treatment of ARDS and lung injury will depend on our capacity to reduce epithelial injury and allow for more rapid restoration of alveolar epithelial integrity and function (Matthay 2014).

## Conflict of Interest

The authors declare that they have no competing interests.

## Supporting information




**Figure S1.** Effect of another anti‐inflammatory drug, methylprednisolone, on edema index after bleomycin‐induced acute lung injury in mice. Wet/dry ratios were measured 3 (*n* = 6, left panel) and 7 days (*n* = 6, right panel) after instillation of saline (Ctl, 0.9%) or bleomycin (Bleo, 4 U/kg) and daily treatments (i.p. administration) with saline (0.9%) or methylprednisolone (methyl, 1 mg/kg, 100 *μ*L). Values are means ± SEM, **P < 0.01 versus Ctl condition, *^∆^P* < 0.05*, ^∆∆^P* < 0.01 versus Dex condition. *n* = 6. 1‐way ANOVA Kruskal–Wallis test and Dunn’s post hoc test.
