# Supplementary figures and images for: Dexamethasone fails to improve bleomycin‐induced acute lung injury in mice
Source: Physiol Rep. 2019 Nov 13;7(21):e14253. doi: 10.14814/phy2.14253 (PMC6854384; doi:10.14814/phy2.14253)

# Supplementary Figure 1

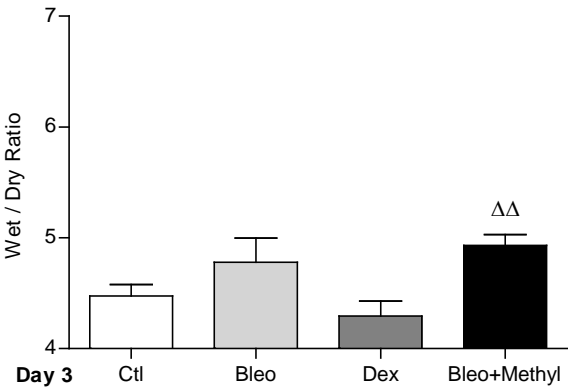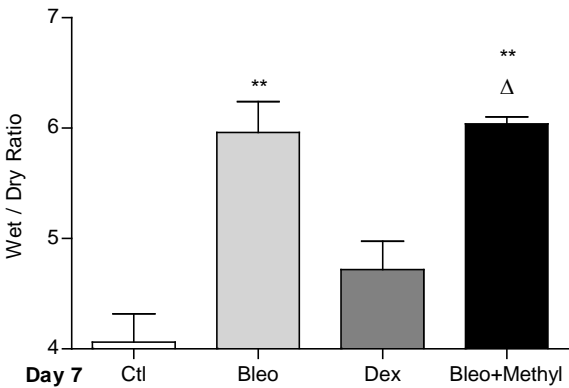

Supplement: Supplementary file 1 — Figure S1. Effect of another anti‐inflammatory drug, methylprednisolone, on edema index after bleomycin‐induced acute lung injury in mice. Wet/dry ratios were measured 3 (n = 6, left panel) and 7 days (n = 6, right panel) after instillation of saline (Ctl, 0.9%) or bleomycin (Bleo, 4 U/kg) and daily treatments (i.p. administration) with saline (0.9%) or methylprednisolone (methyl, 1 mg/kg, 100 μL). Values are means ± SEM, **P < 0.01 versus Ctl condition, ∆P < 0.05, ∆∆P < 0.01 versus Dex condition. n = 6. 1‐way ANOVA Kruskal–Wallis test and Dunn’s post hoc test. [file PHY2-7-e14253-s001.pdf]
